# An increasing role for solvent emissions and implications for future measurements of volatile organic compounds

**DOI:** 10.1098/rsta.2019.0328

**Published:** 2020-09-28

**Authors:** Alastair C. Lewis, Jim R. Hopkins, David C. Carslaw, Jacqueline F. Hamilton, Beth S. Nelson, Gareth Stewart, James Dernie, Neil Passant, Tim Murrells

**Affiliations:** 1 National Centre for Atmospheric Science, University of York, Heslington, York YO10 5DD, UK; 2 Wolfson Atmospheric Chemistry Laboratories, University of York, Heslington, York YO10 5DD, UK; 3 Ricardo Energy and Environment Gemini Building, Fermi Avenue, Harwell, Oxon OX11 0QR, UK

**Keywords:** volatile organic compounds (VOCs), air pollution, atmospheric emissions

## Abstract

Volatile organic compounds (VOCs) are a broad class of air pollutants which act as precursors to tropospheric ozone and secondary organic aerosols. Total UK emissions of anthropogenic VOCs peaked in 1990 at 2,840 kt yr^−1^ and then declined to approximately 810 kt yr^−1^ in 2017 with large reductions in road transport and fugitive fuel emissions. The atmospheric concentrations of many non-methane hydrocarbons (NMHC) in the UK have been observed to fall over this period in broadly similar proportions. The relative contribution to emissions from solvents and industrial processes is estimated to have increased from approximately 35% in 1990 to approximately 63% in 2017. In 1992, UK national monitoring quantified 19 of the 20 most abundant individual anthropogenic VOCs emitted (all were NMHCs), but by 2017 monitoring captured only 13 of the top 20 emitted VOCs. Ethanol is now estimated to be the most important VOC emitted by mass (in 2017 approx. 136 kt yr^−1^ and approx. 16.8% of total emissions) followed by *n*-butane (52.4 kt yr^−1^) and methanol (33.2 kt yr^−1^). Alcohols have grown in significance representing approximately 10% of emissions in 1990 rising to approximately 30% in 2017. The increased role of solvent emissions should now be reflected in European monitoring strategies to verify total VOC emission reduction obligations in the National Emissions Ceiling Directive. Adding ethanol, methanol, formaldehyde, acetone, 2-butanone and 2-propanol to the existing NMHC measurements would provide full coverage of the 20 most significant VOCs emitted on an annual mass basis.

This article is part of a discussion meeting issue ‘Air quality, past present and future’.

## 1. Introduction

The formation of tropospheric ozone from the reactions of volatile organic compounds (VOCs) and NO*_x_* in the presence of sunlight is very well-established science that dates back to atmospheric chemistry research in the 1950s (e.g. [1]). Sunlight in the near-UV either directly destroys VOCs or initiates reactions that generate free radicals that can lead to the oxidation of VOCs, and the generation of peroxy radicals that can convert NO to NO_2_, and thus create a route to the net photo-chemical production of ozone from NO_2_ photolysis. Ozone at the planetary surface has impacts on health that include an exacerbation of asthma [2] and increased risk of death from respiratory causes [3]. More recent advances in the atmospheric chemistry of VOCs have included insight that the oxidation of certain species [4] leads to chemical by-products that can create new particles [5], add mass to the existing particulate matter (PM) [6] or change other properties of PM [7]. There is now a large body of observations confirming the ubiquitous presence of secondary organic aerosol (SOA) in virtually all environments [8]. A consequence is that the environmental motivations for controlling primary anthropogenic VOC emissions now go beyond the established science of ozone formation and form part of PM_2.5_ reduction strategies [9].

The term VOC, used frequently and interchangeably with the longer abbreviation non-methane VOCs (NMVOCs), is a catch-all term for any organic compound found as a gas in the atmosphere. In urban air, NMVOCs can encompass many thousands of different organic compounds including non-methane hydrocarbons (NMHCs), oxygenated, nitrated and halogenated species [10]. Since the term VOC is so broad, for regulatory and policy purposes, more specific definitions are used. One such technical definition is given in official guidelines issued by the European Monitoring and Evaluation Programme (EMEP) and the parallel United Nations Economic Commission for Europe (UNECE) Convention on Long Range Transport of Air Pollution (CLRTAP) for national inventory reporting of emissions. The same definition is also used in the European Commission (EC) Directive 1999/13/EC (Solvent Emissions Directive) [11] and EC National Emissions Ceiling Directive (NECD) [12].

NMVOCs comprise all organic compounds except methane which at 293.15 K show a vapour pressure of at least 0.01 kPa (i.e. 10 Pa) or which show a comparable volatility under the given application conditions.

A slightly different definition of VOCs is given within the 2004/42/EC Paints Directive [13], which refers to a VOC as ‘an organic species with a boiling point less than 250°C at a standard pressure of 101.3 kPa’.

The practical realization of both definitions is that most simple NMHCs with a carbon number falling within the range C_2_ to C_14_ are thought of as VOCs. Longer-chain hydrocarbons (>*n*C_14_) may fall outside of the definition, as may more highly functionalized organic compounds such as organic acids or organic peroxides. The majority of persistent organic pollutants (POPs) have lower vapour pressures than this definition, although some two and three ring polycyclic aromatic hydrocarbons such as naphthalene are considered as VOCs as well as POPs. The EC Air Quality Directive 2008/50/EC [14] includes within it a somewhat broader definition related to an ability to form ozone:

‘volatile organic compounds’ (VOC) shall mean organic compounds from anthropogenic and biogenic sources, other than methane, that are capable of producing photochemical oxidants by reactions with nitrogen oxides in the presence of sunlight

The reporting of emissions as set out in treaties such as NECD and CLRTAP, in principle, takes into account all species that fall within the relevant technical definition. In the case of the UK, this is done through the evaluation of hundreds of different sources where statistical data must be collated for the purposes of reporting a single annual VOC emission total. The majority of countries report totals of emissions from various different sectors. The UK is unusual in then mapping the sectoral emissions onto nearly 700 different species.

Pragmatic decisions are obviously needed with respect to those VOCs that might be measured as part of any verification or regulatory activities. Measurements may be needed to inform directly on attainment of specific health-related VOC concentration targets (for example, for benzene and 1,3-butadiene), or those VOCs that may act as an independent check that estimated emissions changes are being reflected in the ambient atmosphere.

Since all VOC analytical methods have boundaries on specificity and sensitivity, a very limited range of VOCs are typically monitored in ambient air, compared with those actually emitted. Ideally, the species that are routinely monitored in air should reflect those that are indicative of the most significant emissions, and those that cause the greatest harm to health. Across Europe routine monitoring of VOCs has followed guidelines in Annex X of the Directive on Cleaner Air for Europe 2008/50/EC. This recommends the measurement of 31 individual VOCs, all NMHCs, ranging in molecular weight from ethane, through 1,3,5-trimethylbenzene. From these recommendations have followed a range of commercial monitoring instruments based around thermal desorption and the development of traceable standards for calibration to mole and kilogram [15]. The choices around which species have been measured historically has been a mixture of practically (what can be measured) informed by which were the most significant VOCs being emitted when routine monitoring became established in Europe in the 1990s. In the case of the UK, continuous VOC monitoring began around 1992. It is noteworthy, however, that unlike virtually all other air quality parameters covered by the NECD, there is no standard European reference method for measurement, although there have been efforts at standardization associated with calibration and reference materials [16]. This paper evaluates how VOC emissions have changed over the last three decades in terms of both absolute amount and VOC speciation, using the UK as a test case for a high-income country, and the possible implications for future observational VOC networks used to track progress towards emissions targets in 2030 and beyond.

### (a) Evaluating volatile organic compound emission changes using inventories

The National Atmospheric Emissions Inventory (NAEI) estimates UK VOC emissions from anthropogenic sources following methods in the EMEP/EEA Emissions Inventory Guidebook [17] for submission under the revised EU Directive 2016/2284/EU on National Emissions Ceilings (NECD) and the reporting framework of CLRTAP.^1^ Both the NECD and CLRTAP set emissions ceilings with milestone targets for particular dates. For example, the NECD sets a ceiling for 2020 that requires a 32% reduction in total UK emissions relative to 2005 levels, excluding agricultural sources. This equates to 724 kt in 2020 based on the latest NAEI estimates for 2005. The NECD then requires a 39% reduction in emissions relative to 2005 levels by 2030, equivalent to 649 kt.

The NECD and CLRTAP define those VOC sources to be included and excluded from the national inventory (e.g. emissions of NMVOCs from biogenic sources are not included) and the technical definition (see earlier). The Guidebook provides estimation methodologies and default emission factors for each source category, although countries can use country-specific emission factors where these are deemed relevant. Key requirements for inventory reporting are Transparency, Completeness, Consistency, Comparability and Accuracy, and in this respect, it is important to provide a national inventory with time-series consistency going back to at least 1990 and forward to 2030.

The NAEI uses a combination of emission factors from the EMEP/EEA Guidebook and emission rates provided by industry and regulators in the UK. The emission factors represent either (i) the total mass of all individual VOCs when added together or (ii) a metric defined as total hydrocarbons (THCs), a non-speciated mass of VOCs, used, for example, in the case of tailpipe road transport emissions. The speciation of the total emissions into individual VOCs is undertaken separately. Where THC factors are used, the methane emissions calculated separately are subtracted out.

The NAEI uses three basic approaches for estimating total VOC emissions—top down, point source and industry-reported. In the first case, an emission factor approach (top-down) combined with relevant activity statistics is used for many combustion sources including crude oil refineries, other industrial sites and in residential buildings. Factors are also used for transport sources, as well as for some processes in the food and drink industry (for example, bread baking and whisky production) and for some uses of solvents,^2^ including many consumer products.

A point-source approach (bottom-up) can be used where the sum of emissions is estimated/measured and reported by process operators for each emitting site within a sector. The UK estimate is generated simply by summing the emissions reported for all of the sites within a given sector. This approach is used for refinery processes, chemicals industry, oil and gas production and certain types of solvent use in industry such as for printing of flexible packaging, and coating of road vehicles. Key sources for data on point-source emissions are the regulators in England, Scotland, Wales and Northern Ireland who maintain inventories for the processes they regulate, and the BEIS Environmental & Emissions Monitoring System (EEMS) for the offshore oil and gas sector.

In the case of solvent use, the third approach is used for UK emission estimates taking data provided directly by industry. However, this may come with little additional information on emission factors or activity data. Data are often supplied on an *ad hoc* basis and usually cover a limited number of years, and thus, a time-series may need to be generated by splicing together various datasets, possibly from different data providers. Therefore, there is a risk that a time-series might not be fully consistent and estimates for different years may be subject to different levels of uncertainty. However, industry estimates are essential for the VOC inventory: they provide data for sources where the lack of public domain data means that emission factor or point-source approaches cannot be used. Further details of the methods used in the NAEI are provided in the UK's national inventory report submitted annually to the UNECE and NECD. The latest version of the inventory is for years up to 2017 (the 2017 NAEI) as submitted in early 2019 (NAEI, 2019).

### (b) Trends in estimated total volatile organic compound emissions in the UK

**Figure 1.**
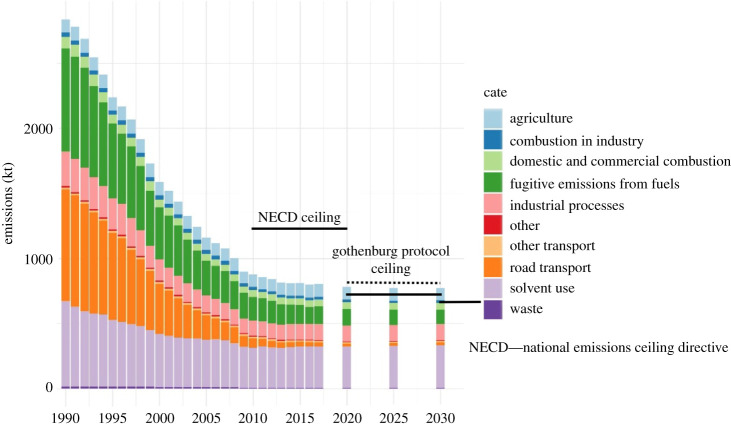
UK emissions of VOCs from anthropogenic sources 1990–2017 and projections for 2020 and 2030. The solid black marker lines represent the NECD ceiling for that time period. The 2020–2029 ceiling is applicable to all the sectors included in the series minus emissions from agriculture (light blue bar). The dotted lines indicate the 2020–2029 ceiling of the revised Gothenburg Protocol and is applicable to all sectors, including agriculture. (Online version in colour.)

Figure 1 shows the trends in anthropogenic VOC emissions from 1990 to 2017 estimated in the NAEI, grouped into 10 major source categories, plus projections for emissions in 2020 and 2030. National emissions are estimated to have decreased from a peak of 2,837 kt in 1990 to 807 kt in 2017, a fall of 72% and a consequence of major reductions from road transport and fugitive emissions from fuels. The decrease in road transport emissions has been mainly due to the introduction of more stringent vehicle emission standards such that by 2017 this sector contributed only approximately 4% of total UK VOC emissions, compared with 30% in 1990.

The largest contribution to VOC emissions in 2017 was from solvents. Overall emissions have decreased for this sector, but by significantly smaller amounts than road transport and fugitive emissions. There have also been relatively smaller reductions in emissions from industrial processes such that this sector contributed 15% of total emissions in 2017; these emissions now mostly come from the food and drink industry which have been increasing since 1990.

**Figure 2.**
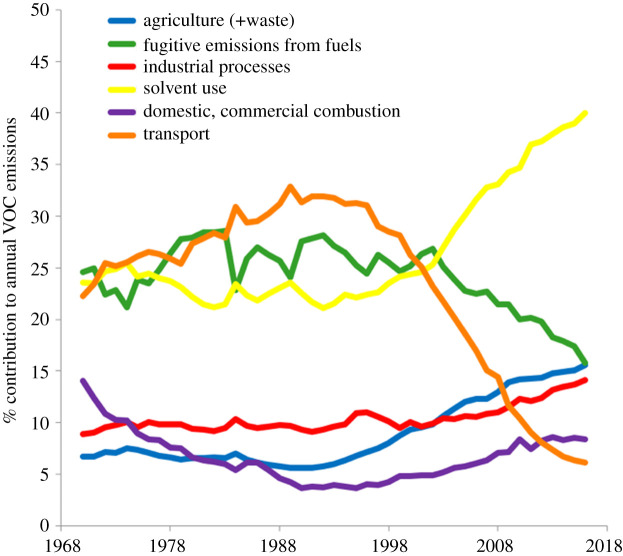
Trends in sectoral contributions to national emissions of VOCs as a percentage of the overall annual national total, 1970–2017, data from uk-air.gov.uk and the National Atmospheric Emissions Inventory. (Online version in colour.)

The period since around 2000 has seen a substantial re-ordering of the relative contributions from each of the major source sectors. Figure 2 shows six sectoral emissions as an annual percentage contribution made to national VOC emissions. There has been a steep decline in the road transport contribution and steady growth since ca. 2000 in the contribution from solvent use, particularly ethanol. Based on the 2017 version of the inventory, the UK met the 2010 NECD emission target of 1,200 kt VOCs and then did so for all subsequent years to 2017 (the last year reported here). While a small further reduction in emissions is anticipated to occur up to 2020, emissions then remain fairly constant up to 2030. This situation arises because emissions from large sources important in the past, such as road transport, have already been reduced significantly, and there remains little scope for further reductions. For example, there are currently no further VOC emission reductions planned beyond the vehicle class of Euro 6/VI. Emissions from sources such as solvent use, food and drink and industrial processes remain fairly flat or increase slightly because emission factors are assumed to remain constant while activities are predicted to remain static or increase slightly (often following population change).

Overall, a small exceedance of the 649 kt NECD target for 2030 is predicted for the UK unless there are further actions to reduce emissions. The size of additional reduction needed to meet the 2030 target is estimated to be approximately 30 kt yr^−1^ by 2030 based on current projections. Meeting those targets is likely to require an increased policy and regulatory focus on those VOCs from the solvent and industrial usage sectors, and so the next section examines which VOC species are significant contributors.

### (c) Volatile organic compound speciation in key emission sectors

There is no statutory requirement to report national inventories of individual VOC species to the NECD or CLRTAP, but a comprehensive speciated inventory is very valuable for other purposes. Given the different chemical reactivities of each component, a speciated inventory is essential for atmospheric models of ozone and SOA formation. It is also necessary for the interpretation of ambient measurements of VOCs and how such information can be used to verify inventory trends.

The UK is one of the few countries to have developed a comprehensive speciated inventory for VOC emissions, and this is widely used in atmospheric modelling. The speciated inventory was first developed in the mid-1990s, but the lack of significant new data sources means that development essentially finished in the early 2000s when the methodology was published [18]. The NAEI's VOC inventory is broken down into 664 species with unique VOC profiles for each emission source. These are mostly as chemically unique species, although occasionally these are expressed as aggregate groups of VOCs, for example, ‘C_13_ aromatics’.

The information used in the development of the species profiles came from various sources, for example industry trade associations in the 1990s provided some speciated estimates including the Solvent Industry Association, the British Coatings Federation and the British Aerosol Manufacturers Association. The solvent industry also helped with speciation of white spirit and other hydrocarbon mixtures. The Environment Agency's Pollution Inventory and similar inventories compiled by other UK regulators include some details of speciation, although the amount of detail is now much less than was the case in the early 1990s. Some analysis was undertaken of fugitive emissions at petrol stations, and species profiles were also provided by the refinery sector. The profiles for vehicle exhaust emissions were taken from the EMEP/EEA Emissions Inventory Guidebook. An important source for other sectors was the US EPA SPECIATE database.

As much of the data used to develop the speciation profiles were gathered during a short period in the late 1990s and early 2000s, and since more recent data are not available, it is assumed that the species emitted by a particular source are the same in all years. While this is likely to be true for sources such as bread baking or gasoline distribution, it is probably not true of technologically evolving sources such as industrial coating processes, chemicals manufacture or the formulation of consumer and household products.

**Figure 3.**
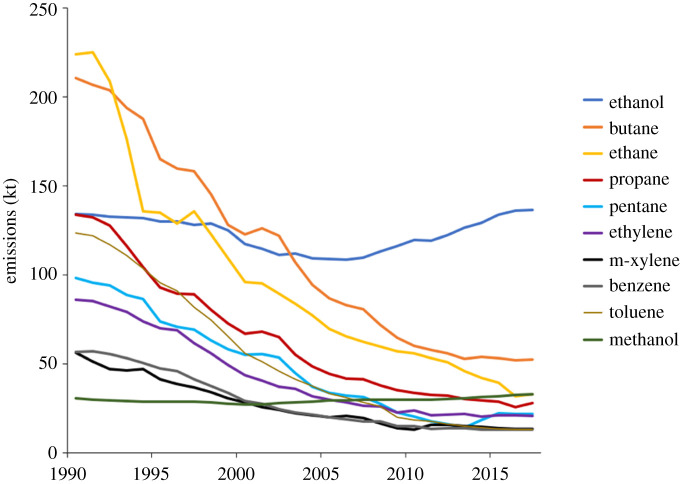
Estimated trends (1990–2017) in the UK emissions of (by rank order in 2017). 1. Ethanol, 2. *n*-butane, 3. methanol, 4. ethane, 5. propane, 6. *n*-pentane, 7. ethylene, 8. *m*-xylene, 9. benzene and 10. toluene. (Online version in colour.)

There are approximately 360 different individual VOC source sectors or processes included within the NAEI, and each of these has a representative chemical speciation associated with it, so it is possible to interrogate the NAEI for annual amounts and therefore emission trends of individual VOCs. Although in total the NAEI contains data on nearly 700 VOCs, a much smaller subset of approximately 40 VOCs represents typically approximately 70% of the total mass of emissions. The 10 most significant individual VOCs in terms of mass emitted represented 45.3% of UK national emissions based on 2017 estimates. Ethanol was the most important VOC comprising 16.8% of all emissions. The recent trends in the 10 highest VOCs by mass emission are shown in figure 3.

Figure 3 highlights a national trend since 1990 of decreasing emissions of simple NMHCs associated with natural gas leakage (ethane), evaporative loss of fuels (e.g. pentane, butane and toluene) and reductions in VOCs from tailpipe emissions (e.g. benzene and ethene). Over the same period, there have been increases in emissions of ethanol and methanol. The increase in ethanol in the NAEI is due to increased reported emissions from the whisky industries and in estimated domestic use of ethanol as a solvent, for example contained within personal care, car care and household products. The more recent addition of bio-ethanol to gasoline is included in the inventory, but due to the relatively low level of contemporary fugitive emissions and exhaust emission, this has not contributed significantly to this upward trend.

Even for simple alkanes there have been some notable changes in the major contributing sources. *n*-butane, for example, is currently the second most abundant VOC in the UK inventory; in 1990, *n*-butane was emitted overwhelmingly from gasoline extraction and fugitive distribution losses (139.8 kt yr^−1^). However, by 2017, the largest anthropogenic source of *n*-butane in the inventory was from its use as an aerosol propellant (25.5 kt yr^−1^) with the gasoline/fugitive losses having been reduced to 23.3 kt yr^−1^.

### (d) Change to emissions of volatile organic compound functional groups

**Figure 4.**
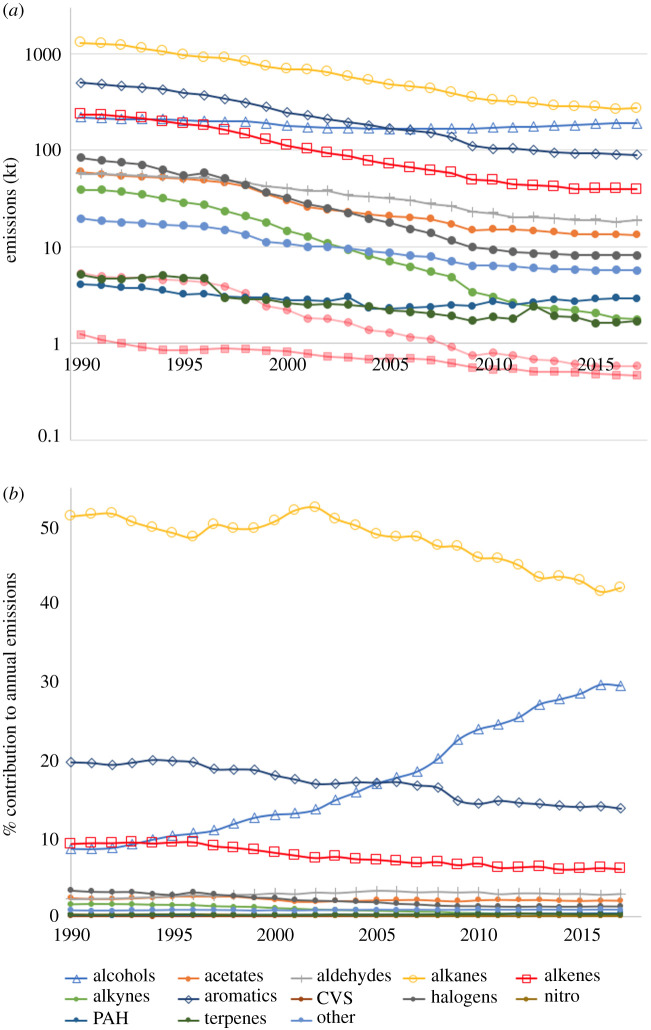
(*a*) Trends in estimated national emissions of functional group classes of VOCs. Contribution of each functional group class to the overall annual national total, 1970 to 2017. (*b*) Contribution of each functional group class expressed as the percentage of annual emissions. Legend common to both plots. (Online version in colour.)

Figure 3 shows the multi-year trends in the estimated emissions of some of the most significant VOCs from a mass-emitted perspective, but it is possible to look more generally at trends in the types of VOCs being emitted, by categorizing the very large number that are individually speciated in the NAEI and then grouping by chemical functional groups. Figure 4*a* shows the estimated emission trends of 12 VOC functional group types, plus a further ‘other’ category for all other minor functionalized VOCs that do not fall within these 12 classes. Figure 4*b* shows the same data but expressed as a group contribution to the annual emissions in percentage terms for each year. The most striking feature is the increased significance of alcohols generally, with significant contributions to 2017 emissions made by 1-propanol, 2-propanol and 1-butanol as well from methanol and ethanol highlighted in the previous figure.

**Figure 5.**
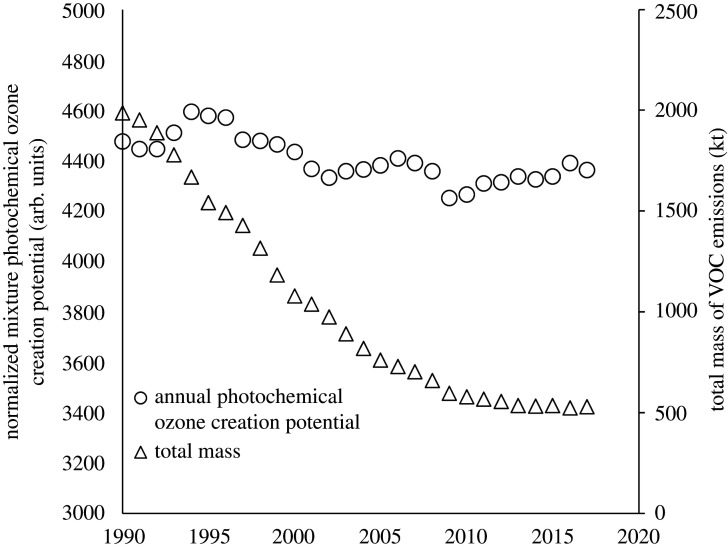
Normalized POCP per average UK unit of VOCs emitted 1990–2017 (left-hand *y*-axis) and total mass of VOCs emitted (right-hand *y-*axis).

Understanding the exact location- and condition-specific impacts of a change in VOC speciation on the tropospheric ozone can only be done fully using explicit modelling and is beyond what we report here. It is possible, however, to evaluate how a change in VOC speciation may impact on the ozone-forming potential of national emissions in the atmosphere by considering the photochemical ozone creation potential (POCP) [19,20] of the VOC mixture. Using the overall POCP of the ensemble of VOCs emitted, it is possible to examine at a bulk level whether the changes in speciation might have led to a change in overall ozone-forming potential, per tonne of ‘average’ UK VOC emissions. Figure 5*a* shows the trends in total VOC emissions for the UK (by mass) and the normalized POCP for each year derived from the top 40 emitted VOCs in that year using Derwent *et al.* [21] POCP values. The 40 VOCs for which the calculation is made represent approximately 70% of overall emissions by mass and assumes that NO*_x_* and all other relevant photochemical parameters are held constant.

Over the 1990–2017 period, the normalized POCP of the UK VOC mixture declined slightly, by approximately 4%. A complex set of changes lie behind this; the largest change in any single VOC is the reduction in ethane emissions (a low POCP compound) and the growth in ethanol (a species of intermediate POCP reactivity). However, there are also concurrent reductions in a range of other high POCP alkenes and aromatic compounds that offset the growth in ethanol. The net change is to an average emission mixture that is very slightly lower in POCP per unit national emissions in 2017 than the mixture being emitted in 1990, but likely too small a change to materially affect ozone formation rates.

### (e) Changes in ambient concentrations

Continuous measurements have been made of a range of non-methane hydrocarbons in the UK Automated Hydrocarbon Network since 1992. The shape of the network has changed over the years, although the methodology has always been based around thermal desorption—GC-FID. Further details can be found on the UK-AIR online resource.^3^ Since 2001, there have been four continuous GC-FID systems measuring NMHCs in the UK and of these the monitoring station at Marylebone Road in Central London has the most complete data record. Although Marylebone Road is formally a roadside location, it does experience the full range of urban emissions given its central position within the city. Other publications have reported data from the UK up to around 2008, for example Von Schneidemesser *et al.* [22]. Here, we show some of the more recent trends in ambient NMHCs as measured at Marylebone Road and then compare to changes indicated in the NAEI.

**Figure 6.**
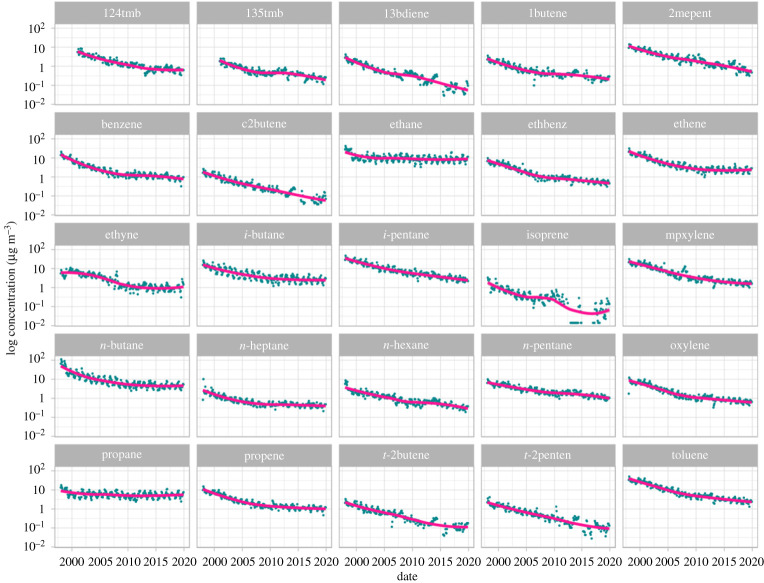
Trends in selected ambient NMHCs measured at the Marylebone Road automated hydrocarbon network station in the centre of London. (Online version in colour.)

Figure 6 shows trends for 25 different species. Each hydrocarbon has a unique behaviour over time, although three distinct trends are observable. For ethane and propane, derived largely from natural gas leakage, there have been only modest reductions in ambient concentrations in central London since 2000. For a range of hydrocarbons derived predominantly from sources such as gasoline evaporation and incomplete combustion, for example xylenes, toluene and 1,3-butadiene, there is a broadly log-linear decrease of approximately two orders of magnitude with reductions being seen up to the most recent year of observations. For some species like butenes, pentenes and 1,3-butadiene, ambient concentrations are now close to instrumental detection limits and there is considerable scatter in the data. There is a third type of behaviour for VOCs that showed initial falls in ambient concentrations in the early 2000s but that have plateaued more recently. These include *i-* and *n-*butane, ethene, benzene, ethyne and propene. This is potentially rationalized as arising from initial falls in concentrations through the reduction of emissions of these species from their road transport source, but that other urban sources also exist that have not declined and that now dominate ambient concentrations.

Ambient concentrations measured at the roadside cannot be expected to directly reflect inventory changes, since roadside monitoring sites of this kind are skewed to detecting changes in the locally dominant road transport source. However, it is still instructive to examine the general scale of reductions seen and the degree of agreement between observation and inventory. Table 1 shows the annual mean concentrations for a range of VOCs and the estimated change in emissions over a similar period. The nature of the Marylebone Road monitoring makes it particularly sensitive to road transport emissions, and for many VOCs, the reductions in roadside concentrations have been greater than those estimated as a percentage of overall national emissions, reflecting particular success in reducing emissions from this sector. In the case of natural gas-derived VOCs like ethane and propane, urban concentrations reported through UK-AIR have changed little in recent years, while national emissions of ethane are estimated to have fallen by 66% between 2000 and 2017 for all sources and by 52% for natural gas leakage according to the NAEI. This mismatch between trends in emission estimates and concentrations may, in part, be rationalized due to the inventory reductions in emissions occurring in remote offshore extraction industries and distribution networks, which are displaced from monitoring sites. It may also be the case that in London, gas leakage rates have been higher than the UK average which the NAEI is based on. It is also important to consider that the lifetimes of these species are long and significant hemispheric background concentrations are present. Observations in Europe have shown similar ambient trends to those reported here, with declines across several European roadside locations [23].

**Table 1.** Selected comparison of emission reductions for individual VOCs estimated in the National Atmospheric Emissions Inventory (2000–2017) and the change in concentrations observed in central London at the Marylebone Road monitoring station.

VOC	2000 annual mean (mg m^−3^)	2018 annual mean (mg m^−3^)	roadside change (%)	2000 emission (kt)	2017 emission (kt)	NAEI change %
ethane	11.5	8.3	−27.6	96.0	33.1	−65.5
propane	6.2	5.2	−16.4	67.1	28.1	−58.1
*i*-butane	10.6	2.4	−77.9	27.8	8.4	−70.0
*n*-butane	22.1	2.9	−86.9	112.8	52.4	−58.5
*i*-pentane	24.8	2.5	−90.1	43.7	21.1	−51.7
benzene	8.1	0.7	−91.9	56.6	13.2	−76.7
toluene	28.0	2.5	−90.1	56.1	13.1	−76.6
ethyl benzene	5.1	0.5	−90.0	22.6	6.3	−72.1
ethene	14.4	2.4	−83.5	43.7	21.1	−51.7
propene	7.0	1.5	−78.5	22.2	5.4	−75.7
1,3-butadiene	1.7	0.3	−93.3	10.5	2.0	−81.4
*t*-2 pentene	1.6	0.1	−93.4	2.9	0.8	−72.4
ethyne	7.0	1.9	−72.0	13.0	1.7	−86.9

**Figure 7.**
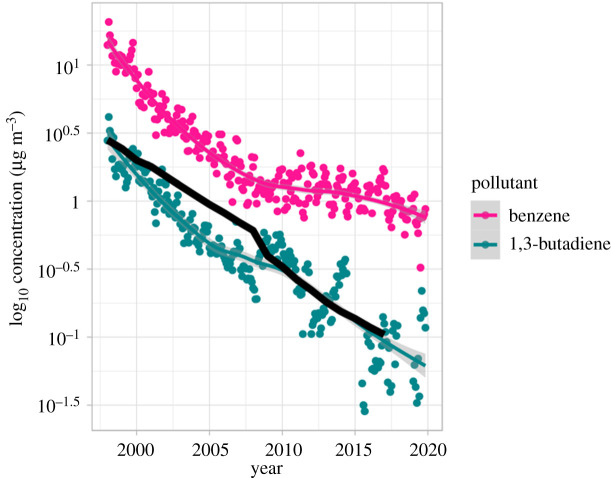
Comparison of trends in roadside 1,3-butadiene and benzene concentrations at Marylebone Road in central London with the National Atmospheric Emissions Inventory estimate of total 1,3-butadiene emissions from the road transport sector (solid black line). (Online version in colour.)

Making direct like-for-like trend comparisons between observations and inventories is generally not appropriate, given multiple contributing sources that define the ambient concentrations of any given VOC. Urban monitoring stations can be sensitive to a particular subset of sources along with reflecting broader patterns of long lifetime VOCs which are influenced by the regional transport. However, 1,3-butadiene is one VOC where some direct comparisons can potentially be made. The lifetime of butadiene during daytime with respect to hydroxyl radical oxidation is very short, around 30 min, and so its measured concentration essentially reflects local sources only. The only major urban source of 1,3-butadiene is thought to be road transport tailpipe emissions. In figure 7, we show the trend in roadside concentrations of 1,3-butadiene (Marylebone Road) against the inventory estimated emissions of 1,3-butadiene from the road transport sector (i.e. excluding all other sources such as refineries and fossil fuel extraction). There is a remarkable degree of agreement in trends between the estimated emissions and ambient roadside observations, with some evidence that for a period, ambient concentrations reduced faster than reported in the inventory, potentially due to overperformance of emission control technologies in the early and mid-2000s. The trends in 1,3-butadiene can be contrasted with benzene, a much longer-lived VOC, which has significantly greater diversity of emission sources beyond road transport tailpipe emissions.

### (f) Impacts of changing speciation on monitoring strategies

Previous sections have shown how at a national level, current emissions in the UK are now significantly influenced by VOCs released from solvent use, both industrial and domestic. As further reductions in emissions are required across Europe, and most reductions from the fossil fuel and transport sectors have likely already been achieved, reducing solvent emissions appear the most feasible route to meet future NECD objectives. Specific policies and interventions that might achieve these aims are beyond this paper, but there will likely be an overarching requirement for ambient observations to continue to provide some external verification that changes in emissions as determined by inventory reporting processes have occurred. This situation creates a measurement challenge since the solvent sector is heavily influenced by oxygenated compounds, rather than NMHCs which current online monitoring infrastructure is generally best configured to detect [24]. There are then further questions raised about where geographically representative measurements should be made if transport sources are no longer significant.

**Figure 8.**
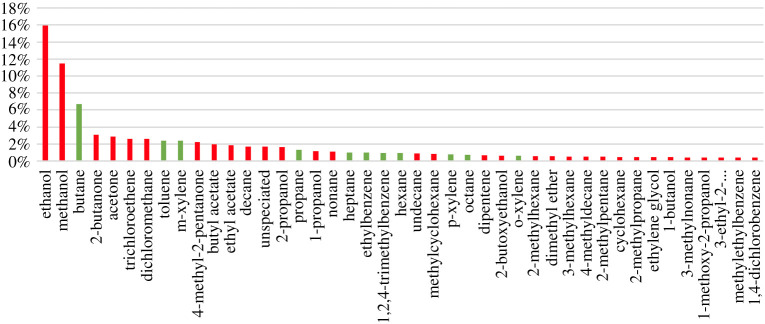
Percentage contribution to the overall emission of VOCs from the ‘solvents and related products' class of emissions in the National Atmospheric Emissions Inventory (2017 edition). Red species are not routinely measured, and green species are included in the UK Defra automated hydrocarbon network. (Online version in colour.)

The current speciation of VOCs from the class of ‘solvents and related products' in the NAEI is likely imperfect due to lack of up to date speciation information from manufacturers, but it provides some guide to the key species that are released. Figure 8 shows the fractional contributions for the most abundant 43 VOCs in this solvent emission class in the NAEI for 2017, dominated again by ethanol and methanol, but with significant contributions from ketones like acetone and 2-butanone and halocarbons including trichloroethene and dichloromethane. The figure is annotated by those species that are currently routinely monitored (in green) with those that are not (in red).

An alternative way of visualizing the impacts of changing emission speciation on monitoring strategies is to consider the most significant VOCs (either individual species or inventory lumped groups) emitted each year and then whether there is coverage in terms of ambient air measurements. We evaluate this by taking a time slice at 5-year intervals from the VOC speciation in the NAEI and rank order the top 40 VOCs emitted in each year.

VOCs in table 2 with a white background are currently measured as part of the UK Automated Hydrocarbon Network, and this is mirrored widely in terms of analytical methodology across Europe and elsewhere. Those species shaded are not currently monitored routinely. It is clear that when the UK/EMEP networks for online monitoring were first designed there was very good alignment between the major VOCs being emitted and the VOCs being monitored. Of the top 20 unique VOCs emitted in 1990, 19 were captured by the Automated Hydrocarbon Network (this excludes a NAEI lumped group of >C_13_). Over time, however, the observational coverage has declined, to 2017 when only 13 of the top 20 VOCs were routinely monitored, including omission of two of the top three most significant VOCs emitted by mass.

**Table 2.** Top 40 anthropogenic VOCs emitted by mass in the UK in different time periods 1990–2017. Bolded species are not routinely measured by existing automated monitoring networks. me, methyl; tmb, trimethylbenzene; TCE, trichloroethylene; DCM, dichloromethane.

rank	1990	1995	2000	2005	2010	2015	2017
1	ethane	butane	butane	**ethanol**	**ethanol**	**ethanol**	**ethanol**
2	butane	ethane	**ethanol**	butane	butane	butane	butane
3	**ethanol**	**ethanol**	ethane	ethane	ethane	ethane	**methanol**
4	propane	toluene	propane	propane	propane	**methanol**	ethane
5	toluene	propane	toluene	pentane	**methanol**	propane	propane
6	pentane	pentane	pentane	toluene	ethylene	pentane	pentane
7	2-me butane	ethylene	ethylene	ethylene	pentane	ethylene	ethylene
8	ethylene	2-me butane	2-me butane	**methanol**	toluene	m-xylene	m-xylene
9	3-me pentane	3-me pentane	3-me pentane	2-me butane	benzene	toluene	benzene
10	2-me propane	benzene	hexane	benzene	2-me butane	benzene	toluene
11	hexane	2-me propane	benzene	m-xylene	m-xylene	2-me butane	**formaldehyde**
12	benzene	hexane	m-xylene	2-me propane	2-me propane	**formaldehyde**	2-me butane
13	m-xylene	m-xylene	2-me propane	hexane	hexane	hexane	hexane
14	ethylbenzene	propylene	**methanol**	**formaldehyde**	**formaldehyde**	2-me propane	**decane**
15	propylene	ethylbenzene	ethylbenzene	heptane	**decane**	**decane**	**acetone**
16	o-xylene	3-me pentane	propylene	propylene	**acetone**	**acetone**	2-me propane
17	3-me pentane	o-xylene	heptane	ethylbenzene	**2-butanone**	**2-butanone**	**2-butanone**
18	heptane	**other, C>13**	**formaldehyde**	**acetone**	propylene	ethylbenzene	1,2,4-tmb
19	**other, C>13**	m & p-xylene	2-me pentane	decane	heptane	1,2,4-tmb	ethylbenzene
20	m & p-xylene	**methanol**	**TCE**	1,2,4-tmb	**2-propanol**	**2-propanol**	**2-propanol**
21	acetylene	heptane	o-xylene	3-me-pentane	1,2,4-tmb	heptane	C7 alkanes
22	**TCE**	**formaldehyde**	octane	**2-butanone**	ethylbenzene	propylene	propylene
23	**methanol**	acetylene	1,2,4-tmb	octane	3-me pentane	**ethyl acetate**	heptane
24	octane	**acetone**	**other, C>13**	o-xylene	octane	C7 alkanes	C8 alkanes
25	2-me propene	2-me propene	**acetone**	2-me pentane	2-me pentane	3-me pentane	**ethyl acetate**
26	**formaldehyde**	1,2,4-tmb	m & p-xylene	**TCE**	o-xylene	**nonane**	3-me pentane
27	1,2,4-tmb	**TCE**	acetylene	**2-propanol**	**nonane**	**undecane**	**undecane**
28	**acetone**	octane	**2-butanone**	**unspeciated**	**undecane**	o-xylene	**nonane**
29	**DCM**	**methyl acetate**	**decane**	**other, C>13**	C7 alkanes	**me-pentanone**	**me-pentanone**
30	**111-TCethane**	**C9 aromatics**	2-me propene	m & p-xylene	**ethyl acetate**	octane	2-me pentane
31	**methyl acetate**	**2-butanone**	**C13+ aromatic**	**C13+ aromatic**	**me-pentanone**	2-me-pentane	o-xylene
32	**C13+ aromatic**	C7 alkanes	C7 alkanes	2-me propene	**C13+ aromatic**	**C8 alkanes**	octane
33	**2-butanone**	**decane**	**2-propanol**	**C8 alkanes**	**C8 alkanes**	**p-xylene**	p-xylene
34	**decane**	**C8 alkanes**	**unspeciated**	**me-pentanone**	**butyl acetate**	**butyl acetate**	**butyl acetate**
35	C7 alkanes	**2-propanol**	**methyl acetate**	**nonane**	**DCM**	**1-butanol**	**DCM**
36	**2-propanol**	**ethyl acetate**	**C8 alkanes**	acetylene	2-me propene	**DCM**	**1-butanol**
37	**C8 alkanes**	**DCM**	**DCM**	**DCM**	p-xylene	**C13+ aromatic**	**1-propanol**
38	**ethyl acetate**	**me-pentanone**	**ethyl acetate**	**undecane**	**unspeciated**	**1-propanol**	**C13+ aromatic**
39	**me-pentanone**	**unspeciated**	**me-pentanone**	**butyl acetate**	**TCE**	**TCE**	**TCE**
40	**1-butanol**	**111-TCethane**	**butyl acetate**	**ethyl acetate**	**1-butanol**	**C3-benzene**	**C3-benzene**

It is also instructive to note those VOCs which are not as significant as they once were. Ethyne is one of the most challenging VOCs to measure using thermal desorption techniques due to its very low boiling point, and this can be a defining factor for much of the analytical instrumentation for analysis (for example, breakthrough volume). By 2017, ethyne had fallen to 82nd when ranked by mass of emissions from a position in 1990 of 21st. An argument can also be made that the various isomers of butenes and pentenes that were once important as contributors to both total mass emissions and to reactivity and ozone creation potential in the 1990s have been reduced so significantly that the effort expended on their measurement in networks may not be particularly productive.

### (g) Global applicability to emissions and monitoring in other locations

Since there is no standardized international requirement to report speciated VOCs as part of emissions control treaties, it is difficult to make direct comparisons between the UK-specific conclusions we report here and other global locations. We make some attempt to reality-check whether the influence of oxygenated VOCs is as great as suggested by UK inventories by examining the most significant VOCs detected in ambient air in the UK and in other countries, using data collected from short periods of research observations and process studies. Ambient data are, of course, not directly comparable to emission inventories at a national scale, since large point sources may not be detected at a given measurement location and there may be important seasonal factors not captured in short-term measurements that are reflected in annual averages. Many oxygenated VOCs are also produced through atmospheric oxidation, so they may be elevated in ambient air not solely because of direct anthropogenic emission, but also as a by-product of the degradation of other VOCs. This is particularly important for acetaldehyde and formaldehyde which are common degradation products, including also from natural emissions such as isoprene. We exclude those from this analysis. From the recent literature, we note the work of MacDonald *et al.* [25] which shows ethanol, iso-propanol and acetone as three of the four most abundant individual VOCs measured in ambient air in Pasadena, USA. From our own observations made using two-column GC-FID in various locations, we find a good degree of consistency between the top VOCs ranked by ambient concentrations in different global locations. Table 3 shows examples of the most abundant VOCs in ambient air in London, Beijing (measured during wintertime) and Delhi (during the post-monsoon period), all part of research intensive observation periods. We use wintertime data since this is a closer reflection of source emission profiles since oxidation losses are lower than in summer. The concentration of VOCs found in each location differs very significantly, with order magnitude differences between London (lowest) and Delhi (highest). However, for all three urban locations, ethanol is the most abundant VOC with methanol and acetone also found in the top 10 species in all three locations.

**Table 3.** Most abundant VOCs ranked by concentrations based on the recent research of wintertime city centre observations in London [26], Beijing [27] and Delhi all based on a common two-channel gas chromatography–flame ionization detection method.

rank order	London monthly mean (µg m^−3^)	Beijing monthly mean (µg m^−3^)	Delhi monthly mean (µg m^−3^)
year	2012	2016	2018
period	Jan	Nov–Dec	Oct–Nov
1	ethanol (10.7)	ethanol (23.7)	ethanol (72.4)
2	ethane (8.3)	acetone^a^ (19.6)	*n*-butane (60.8)
3	acetone^a^ (8.1)	*m* + *p*-xylene (17.7)	methanol^a^ (53.7)
4	methanol^a^ (6.8)	methanol^a^ (13.2)	propane (48.5)
5	*n*-butane (5.2)	propane (13.1)	iso-butane (32.3)
6	propane (4.9)	ethane (11.8)	toluene (30.4)
7	iso-butane (2.6)	ethene (9.4)	iso-pentane (28.8)
8	iso-pentane (3.5)	toluene (7.8)	acetone^a^ (25.3)
9	toluene (2.6)	*n*-butane (7.0)	ethane (23.1)
10	butanol (2.5)	*i*-pentane (6.5)	*m* + *p*-xylene (15.1)

^a^Minor contribution to ambient concentrations possible from the oxidation of other VOCs.

We note that since research observations exist for many oxygenated VOCs, it demonstrates that there are no insurmountable technical obstacles to improving routine measurement coverage [28]. This paper does not make recommendations for how best a broadened set of VOCs might be measured in automated networks, but there are several proven analytical approaches available—widely reported in the literature are GC-FID [29], GC–MS [30] and online chemical ionization mass spectrometry [31]. Indeed, the World Meteorological Organization Global Atmosphere Watch (GAW) programme has already supported a set of calibration infrastructure and measurement guidelines for some oxygenated VOCs [32], although measurements remain sparse at GAW background stations [33].

## 2. Conclusion

Inventory estimates of total VOC emissions in the UK show significant decreases over the past 30 years from a peak around 1990, which is mirrored in ambient measurements of some NMHCs. The most significant sources in the 1990s were NMHCs related to fossil fuel usage, in particular from natural gas extraction and distribution, gasoline tailpipe emissions and fugitive fuel losses. Policies and regulations across multiple sectors to reduce emissions have been very effective, particularly from road transport, which now contributes only a small amount (approx. 4%) to total UK VOC emissions. There has been little change in overall emissions from industrial and domestic solvent usage, and this has resulted in a growth of this sector as a proportion of national emissions. Along with this change has been a change in both the relative and absolute amounts of individual VOCs emitted. Ethanol is now the most abundant VOC emitted in the UK and overall, short-chain alcohols are the most important functional group, measured by mass. The shift in functional groups has not, however, had an appreciable impact on overall average POCP (a 4% decline) since the growth in ethanol has been balanced by losses in reactivity in other species, notably alkenes and mono-aromatics.

If future changes in emissions are to be independently tested against external atmospheric monitoring, then a revised analytical strategy is needed, both in terms of species quantified and locations of the measurement themselves. The current online VOC networks used across Europe predominantly focus on the measurement of NMHCs, with only rather limited coverage of certain functionalized VOCs using off-line methods, for example to measure aldehydes. This results in many of the major oxygenated VOCs emissions going undetected and skewed in geography towards VOCs emitted from transport sources. Without some change to this position, it will not be possible to evaluate how successful future policies and technical interventions have been in reducing solvent emissions, noting that reductions in VOCs are needed in many countries to meet NECD and CLRTAP obligations for 2030. The addition of ethanol, methanol, formaldehyde, acetone, 2-butanone and 2-propanol to the existing suite of NMHC measurements would provide for a full observational coverage of the 20 most significant VOCs emitted on an annual mass basis. It may be possible for a change in future observational strategy to be brought closer to cost-neutral in operational terms by ceasing observations of certain analytically challenging non-methane hydrocarbons, such as acetylene, butenes and pentenes, which have seen their emissions fall very significantly and that are now often close to or below instrumental detection limits in UK ambient air.

## Footnotes

1 See https://www.ceip.at/reporting-instructions for reporting requirements of estimating and reporting emissions data under the CLRTAP.

2 The term ‘solvents’ is rather imprecise and could be possibly interpreted as defining some specific chemical functionality or property, although there is no universally agreed definition. In this paper, when we refer to solvents we are referring to those VOCs included in that emissions class for the purposes of international emissions reporting. In a chemistry laboratory setting, the term solvents bring to mind VOCs such as acetone, ethanol, toluene and so on, and they are indeed major species in solvent inventories. For emissions reporting purposes, a wider range of VOCs are included under the definition including aerosol propellants such as butane and some long-chain aliphatic compounds.

3 See https://uk-air.defra.gov.uk/networks/network-info?view=hc for further details of the UK Automatic Hydrocarbon Network.

## Data accessibility

All data in this study are available for free download from public repositories. Data on atmospheric VOC emissions are included in the National Atmospheric Emissions Inventory available at the uk-air.gov.uk website. Ambient monitoring data from the Defra Automatic Hydrocarbon network are also available for download from uk-air. Data on which mean concentrations of VOC in London and Beijing (table 3) are based are available from the Centre for Environment Data and Analysis: www.ceda.ac.uk. Data on VOCs in Delhi are currently subject to embargo but will be available on CEDA in 2021.
